# Meropenem-Responsive Pandoraea apista Pneumonia Despite High-Level In Vitro Resistance: A Rare Case in an Older Non-cystic Fibrosis Patient

**DOI:** 10.7759/cureus.99167

**Published:** 2025-12-13

**Authors:** Mohamed Abdelgalil, Abdalla Diyab

**Affiliations:** 1 Critical Care Medicine, Salma Rehabilitation Hospital, Abu Dhabi, ARE

**Keywords:** antibiotic, aspiration, cystic fibrosis, pandoraea, sepsis

## Abstract

*Pandoraea apista* is a rare, non-fermenting, gram-negative bacillus mainly seen in cystic fibrosis (CF) patients. It is now increasingly being isolated in non-CF patients, mostly those who are critically ill or have multiple comorbidities. We describe a patient with no history of CF who was admitted with hospital-acquired pneumonia. The sputum culture grew *P. apista*, which was resistant to many antibiotics, including meropenem. Despite the in vitro resistance, the patient improved when kept on meropenem after the culture result was available. This was mostly due to optimized dosing and favorable exposure above the minimum inhibitory concentration. Our case highlights these diagnostic challenges and variable resistance patterns. It also emphasizes the important role of personalized therapy tailored to each patient and their condition.

## Introduction

*Pandoraea apista*, from the genus *Pandoraea*, first described in 2000, is primarily associated with cystic fibrosis (CF). This genus consists of aerobic or facultatively anaerobic, gram-negative bacteria. Characteristic features include unipolar flagellar motility, nitrate reduction, and lactose fermentation [1].

*Pandoraea* is an environmental contaminant found in soil and water. Species isolated from humans include *P. apista*, *Pandoraea pulmonicola*, *Pandoraea pneumoniae*, *Pandoraea sputorum*, and *Pandoraea norimbergensis*. When identified using routine testing, these species can be misidentified as *Ralstonia* or *Burkholderia* in standard biochemical tests [2].

*Pandoraea* is increasingly seen in patients with CF and has the potential to cause chronic respiratory infections, associated with decreased lung function and more frequent episodes of exacerbation. This infection is often linked with colonization by other organisms, most commonly *Pseudomonas aeruginosa*. It has been observed that *Pandoraea* can cause invasive disease, because many respiratory infections result in bacteremia [3].

To our knowledge, there are currently no defined breakpoints regarding antimicrobial susceptibility testing, which is why there is no universally accepted optimal treatment plan. The species are generally resistant to most antibiotics, with variations in susceptibility to piperacillin-tazobactam, aminoglycosides, and fluoroquinolones. This organism is often resistant to meropenem while remaining sensitive to imipenem [4].

Our case is unique because the patient is a non-CF individual with no underlying lung disease who responded to meropenem despite showing in vitro resistance.

## Case presentation

An 87-year-old Middle Eastern man, weighing 42 kg, with a background of type 2 diabetes mellitus, hypertension, stage 4 chronic kidney disease, dyslipidemia, and a previous bilateral posterior cerebral artery stroke resulting in dysphagia and aspiration risk (nasogastric tube-fed and bedridden), was admitted from a long-term care and post-acute rehabilitation unit with fever. He was started empirically on intravenous antibiotics and fluids, and a central venous catheter was inserted for resuscitation. Due to worsening respiratory status, he was transferred to the ICU for further management.

**Figure 1:**
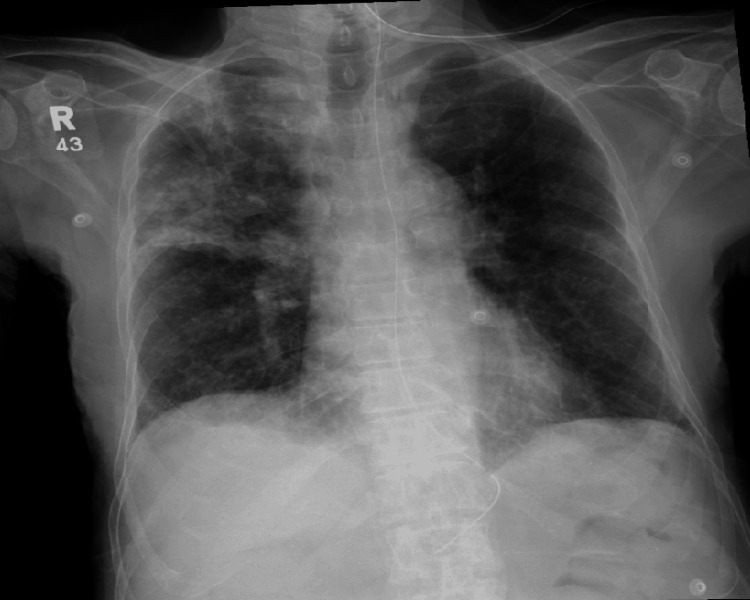
Chest X-ray showing opacity in the right lung

In the ICU, the patient presented with acute hypoxemic respiratory failure secondary to hospital-acquired pneumonia, with right-sided infiltrates on chest X-ray (Figure 1). He was placed on high-flow nasal cannula (HFNC) and started on meropenem. On admission, he was febrile, hypotensive, tachycardic, and tachypneic, with a Glasgow Coma Scale score of 10. He was resuscitated, stabilized, and showed clinical improvement by the following day, allowing transfer back to the ward and continued meropenem therapy.

Laboratory investigations revealed leucocytosis and markedly elevated inflammatory markers. His C-reactive protein (CRP) kept increasing on the day of ICU admission, and then started decreasing after clinical improvement and response to meropenem over the course of a few days. This was followed by improvement of all the other markers (Table 1), indicating a satisfactory response to meropenem.

**Table 1:** Trends in key inflammatory markers during hospitalization WBC, White Blood Cell Count; CRP, C-Reactive Protein; PCT, Procalcitonin

-	Day 1	Day 3	Day 5	Day 7	Day 10	Reference Range
WBC	7.5	15.3	10.5	8.8	-	4.0 - 10.0 × 10⁹/L
CRP	185	373	268	171	-	<5 mg/L
PCT	25	-	-	-	0.46	<0.5 ng/mL

Microbiological studies came back, and a tracheal aspirate culture grew *P. apista* and *Citrobacter koseri*. *P. apista* demonstrated resistance to multiple β-lactams, with intermediate susceptibility to meropenem (Table 2), while *C. koseri* was sensitive to most tested agents, including meropenem and piperacillin-tazobactam. Blood and urine cultures were negative. The patient was continued on meropenem for a total of seven days, during which he became afebrile, inflammatory markers improved, and repeat sputum cultures were negative.

**Table 2:** Sputum culture and sensitivity showing Pandoraea apista TMP-SMX, Trimethoprim-Sulfamethoxazole; MIC, Minimum Inhibitory Concentration

Specimen type	Sputum	
Organism isolated	Pandoraea apista	
Antibiotic	Susceptibility	MIC value
TMP-SMX	Sensitive	-
Minocycline	Sensitive	-
Ceftazidime	Resistant	-
Cefepime	Sensitive	-
Meropenem	Resistant	≥32 µg/mL

Notably, the patient was maintained on meropenem at a dose of 1 g every 12 hours, despite the clinical pharmacy recommendation to reduce it to 500 mg twice daily in view of his renal impairment. This higher dosing may have contributed to the favorable outcome by achieving adequate drug exposure above the minimum inhibitory concentration (MIC) for a sufficient duration of the dosing interval, consistent with the time-dependent killing characteristic of β-lactam antibiotics. The patient ultimately achieved both microbiological and clinical recovery.

## Discussion

*Pandoraea* species were first identified and classified by Coenye et al. in Belgium in 2000. The genus *Pandoraea* includes five named species (*P. apista*, *P. pulmonicola*, *P. pnomenusa*, *P. sputorum*, and *P. norimbergensis*) and four unnamed genomospecies [3]. These bacteria are non-fermenting, gram-negative rods that frequently exhibit multidrug resistance and are typically isolated from patients with CF, as well as from environmental sources such as soil and water [4].

Although human infections caused by *Pandoraea* species have been infrequently documented, the regions where such cases have been reported - primarily Europe, the United States, and Australia - reflect the geographic distribution of CF populations. Furthermore, only a limited number of reports have detailed infections attributable to this emerging opportunistic pathogen in individuals without CF [4].

*P. apista* is a rare cause of pneumonia in individuals without CF. In a systematic review encompassing 43 reported cases of *Pandoraea* infections, 74% of patients presented with pneumonia, while 39% of the overall cohort had underlying CF [5]. Critical illness, invasive ventilation, and previous antibiotic exposure are common among non-CF patients. Despite its rarity, it is essential to acknowledge that *Pandoraea* can present as a hospital-acquired infection, as illustrated in our case.

Standard microbiological testing has limitations and may lack accuracy; consequently, *Pandoraea* species are often misidentified as *Ralstonia*, *Burkholderia*, or *Stenotrophomonas* species. Therefore, the prevalence of *Pandoraea* spp. may be underestimated. In alignment with previous reports, the organism in our case exhibited resistance to several antibiotics, including piperacillin-tazobactam and meropenem. Notably, our patient responded to meropenem despite the resistance indicated in the susceptibility report [4].

In our Kirby-Bauer testing, the isolate was sensitive to minocycline, trimethoprim-sulfamethoxazole (TMP-SMX), and cefepime, while demonstrating resistance to meropenem and ceftazidime. This finding partially correlates with the literature: Dlewati et al. reported that their *P. apista* case was sensitive to amikacin, fluoroquinolones, imipenem, and minocycline, while resistant to cefepime, ceftazidime, and meropenem [6]. In Itoh et al.’s case of *P. apista* bacteremia, the isolate was susceptible to TMP-SMX, and a positive clinical response to this treatment was documented [4]. This underscores the challenges in predicting susceptibility, emphasizing that treatment should always be guided by culture and sensitivity results.

Geremia and Di Bella reported that most *P. apista* isolates exhibit resistance to various common antibiotics, including β-lactams, aminoglycosides, fluoroquinolones, and polymyxins. Notably, many strains remain sensitive to imipenem despite resistance to meropenem; this phenomenon is attributed to the production of specific oxacillinase-type β-lactamases (OXA), particularly OXA-1152 [7].

Our isolate demonstrated resistance to meropenem, with a very high MIC of 32 µg/mL. This finding aligns with numerous literature reports indicating that *P. apista* frequently exhibits high-level meropenem resistance [6,8]. Although not tested in our case, imipenem is often more effective against *P. apista*, with an in vitro sensitivity rate of 83% [4]. This discrepancy can be explained by the preferential hydrolysis of meropenem by *Pandoraea* oxacillinases compared to imipenem, which clarifies the MIC pattern observed in our case and others.

Despite the high-level resistance to meropenem, our patient has shown clinical improvement while receiving this medication. This observation is noteworthy and may be attributed to a pharmacokinetic and pharmacodynamic effect that allowed drug levels to exceed the MIC. It is plausible that a higher-than-usual dose facilitated the achievement of desired levels, because β-lactams exert their bactericidal effect by maintaining concentrations above the MIC for a specified duration during the dosing interval [9]. In the context of chronic kidney disease, the higher administered dose and reduced clearance may have contributed to prolonged drug exposure above the MIC [9,10]. Impaired renal clearance, combined with the high dose used and effective drug penetration in lung tissue, likely facilitated the attainment of adequate drug concentrations.

It is unlikely that this case represents colonization, because the previous culture did not reveal *Pandoraea*, and the observed growth was moderate compared to the light growth of *C. koseri*, indicating that the latter was a colonizer.

It is important to note that cefepime, although sensitive in our case, is not universally effective; some reports have documented intermediate or resistant outcomes [5]. Despite the resistance, our patient experienced clinical improvement following meropenem treatment, supporting the notion that *Pandoraea*’s response to antibiotics can be unpredictable. Unmeasured pharmacokinetic effects, or factors related to host immune recovery, may have also contributed to bacterial clearance.

Similar clinical-microbiological discordance (clinical success despite high in vitro resistance) has been documented in *Pandoraea* and other non-fermenting, gram-negative bacilli, indicating that host factors, tissue penetration, and optimized dosing may counteract apparent resistance [11].

This case underscores the necessity of considering *P. apista* in nosocomial pneumonia cases unresponsive to standard therapy. Following the identification of *P. apista*, it is imperative to adjust antibiotic regimens to a targeted approach. Infections caused by *Pandoraea* are associated with significant morbidity, particularly when inappropriate antibiotics are administered.

The management of this genus is complicated by their resistance mechanisms - both intrinsic and acquired - as well as the absence of standardized susceptibility breakpoints for antibiotics. Among the available treatments, imipenem and TMP-SMX remain the primary options for antibiotic therapy [7]. Further research is required to develop internationally standardized treatment protocols for these increasingly prevalent organisms.

## Conclusions

*P. apista* is a rare organism that is being increasingly identified in non-CF patients, especially those who are critically ill and at risk of hospital-acquired infections. This case highlights the challenges inherent in diagnosing and treating this organism, including resistance to multiple antibiotic classes and the limitations of standard identification methods. Our patient responded to meropenem despite a high MIC, suggesting that, under certain circumstances - such as optimized drug exposure - clinical response may not align with in vitro resistance. This emphasizes the importance of individualized clinical judgment, as well as considerations of pharmacodynamics and pharmacokinetics, in conjunction with close multidisciplinary collaboration in managing infections caused by such organisms.

In instances of nosocomial infections that do not respond to standard therapy, *Pandoraea* should be considered as a potential causative agent during the formulation of treatment strategies. Treatment should be guided by accurate microbiological identification and susceptibility testing whenever feasible. Therapeutic drug monitoring may play a crucial role in the management of carbapenems, reflecting current trends in addressing gram-negative bacteria. Ongoing reporting of such cases is essential for refining future diagnostic and therapeutic strategies for this increasingly emergent opportunistic pathogen.

## References

[1] Li L, Zhang Y, He F, Wu N (2025). Genomic insights into a multidrug-resistant pandoraea apista clinical isolate carrying bla(OXA-153) from China. J Glob Antimicrob Resist.

[2] Singh S, Sahu C, Patel SS, Garg A, Ghoshal U (2021). Pandoraea apista bacteremia in a COVID-positive man: a rare coinfection case report from North India. J Lab Physicians.

[3] Coenye T, Liu L, Vandamme P, LiPuma JJ (2001). Identification of Pandoraea species by 16S ribosomal DNA-based PCR assays. J Clin Microbiol.

[4] Itoh N, Akazawa N, Ishibana Y, Masuishi T, Nakata A, Murakami H (2022). Clinical and microbiological features of obstructive cholangitis with bloodstream infection caused by Pandoraea apista identified by MALDI-TOF mass spectrometry and ribosomal RNA sequencing in a cancer patient. BMC Infect Dis.

[5] Ziogou A, Giannakodimos A, Giannakodimos I, Tsantes AG, Ioannou P (2024). Pandoraea infections in humans - a systematic review. J Clin Med.

[6] Dlewati MM, Aung PP, Park K, Rodriguez JA, Poon KK (2021). Meropenem-resistant Pandoraea pneumonia in a critically ill patient with COVID-19. Cureus.

[7] Geremia N, Di Bella S (2025). Managing multidrug-resistant Pandoraea spp.: current evidence and knowledge gaps. Infez Med.

[8] Lin C, Luo N, Xu Q, Zhang J, Cai M, Zheng G, Yang P (2019). Pneumonia due to Pandoraea apista after evacuation of traumatic intracranial hematomas: a case report and literature review. BMC Infect Dis.

[9] Cojutti P, Sartor A, Righi E, Scarparo C, Bassetti M, Pea F (2017). Population pharmacokinetics of high-dose continuous-infusion meropenem and considerations for use in the treatment of infections due to KPC-producing Klebsiella pneumoniae. Antimicrob Agents Chemother.

[10] Benítez-Cano A, Luque S, Sorlí L (2020). Intrapulmonary concentrations of meropenem administered by continuous infusion in critically ill patients with nosocomial pneumonia: a randomized pharmacokinetic trial. Crit Care.

[11] Kruis T, Menzel P, Schwarzer R (2023). Outbreak of Pandoraea commovens among non-cystic fibrosis intensive care patients, Germany, 2019-2021. Emerg Infect Dis.

